# Adenoviral targeting of malignant melanoma for fluorescence-guided surgery prevents recurrence in orthotopic nude-mouse models

**DOI:** 10.18632/oncotarget.6670

**Published:** 2015-12-18

**Authors:** Shuya Yano, Kiyoto Takehara, Hiroyuki Kishimoto, Yasuo Urata, Shunsuke Kagawa, Michael Bouvet, Toshiyoshi Fujiwara, Robert M. Hoffman

**Affiliations:** ^1^ AntiCancer, Inc., San Diego, CA, USA; ^2^ Department of Surgery, University of California San Diego, La Jolla, CA, USA; ^3^ Department of Gastroenterological Surgery, Okayama University, Graduate School of Medicine, Dentistry and Pharmaceutical Sciences, Okayama, Japan; ^4^ Oncolys BioPharm Inc., Tokyo, Japan

**Keywords:** melanoma, nude mice, fluorescence-guided surgery (FGS), adenovirus, OBP-401

## Abstract

Malignant melanoma requires precise resection in order to avoid metastatic recurrence. We report here that the telomerase-dependent, green fluorescent protein (GFP)-containing adenovirus OBP-401 could label malignant melanoma with GFP *in situ* in orthotopic mouse models. OBP-401-based fluorescence-guided surgery (FGS) resulted in the complete resection of malignant melanoma in the orthotopic models, where conventional bright-light surgery (BLS) could not. High-dose administration of OBP-401 enabled FGS without residual cancer cells or recurrence, due to its dual effect of cancer-cell labeling with GFP and killing.

## INTRODUCTION

Despite recent advances, the current therapeutic approaches in melanoma are not satisfactory and intensive research in this area is still required [1].

Cancer surgery requires precise identification of tumor margins. Green fluorescent protein (GFP) fluorescence can intensely illuminate even single cells *in vivo* [2, 3]. Introducing and selectively activating the GFP gene in tumors *in vivo* was made possible by the development of OBP-401, a telomerase-dependent, replication-competent adenovirus expressing GFP. In an intraperitoneal nude mouse model of disseminated human colon cancer, fluorescence-guided surgery (FGS) enabled resection of tumor nodules labeled with GFP by OBP-401 [4]. Recurrent tumor nodules brightly expressed GFP, indicating that initial OBP-401-GFP labeling of peritoneal disease was genetically stable [5].

Glioblastoma multiforme (GBM) is one of the most invasive of cancers and is not totally resectable using standard bright-light surgery (BLS) or current FGS strategies. We previously developed a curative strategy for FGS of GBM using high-dose OBP-401 to selectively label GBM with GFP. OBP-401-based FGS enabled curative resection of GBM without recurrence for at least 150 days, compared to less than 30 days with BLS [6].

OBP-401-based FGS resulted in superior resection of soft-tissue sarcoma (STS) in an orthotopic nude mouse model, compared to BLS. High-dose OBP-401 enabled FGS without residual sarcoma cells or local or metastatic recurrence, due to its dual effect of cancer-cell labeling with GFP and killing. High-dose OBP-401 based-FGS improved disease-free survival as well as preserved muscle function compared with BLS [7].

OBP-401 was used to label the cancer cells of a pancreatic cancer patient-derived orthotopic xenograft (PDOX) nude mouse model. The PDOX was previously grown in a red fluorescent protein (RFP) transgenic mouse that stably labeled the PDOX stroma cells bright red. The color-coded PDOX model enabled FGS to completely resect the pancreatic tumors including stroma. Dual-colored FGS significantly prevented local recurrence, which bright-light surgery or single-color FGS could not. FGS, with color-coded cancer and stroma cells has important potential for improving the outcome of recalcitrant-cancer surgery [8].

OBP-401-FGS of tumors in the lung enabled complete lung tumor resection with no residual fluorescent tumor [9].

OBP-401 tumor illumination enabled effective FGS of an orthotopic mouse model of human osteosarcoma model as well as eradication of residual osteosarcoma cells after BLS. OBP-401-FGS significantly inhibited local recurrence and lung metastasis after surgery thereby prolonging survival [10].

In the present report, we demonstrate that OBP-401 GFP labeling *in situ* enables precise FGS of malignant melanoma without recurrence.

## RESULTS AND DISCUSSION

### OBP-401 targets malignant melanoma cell lines with GFP *in vitro*

**Figure 1:**
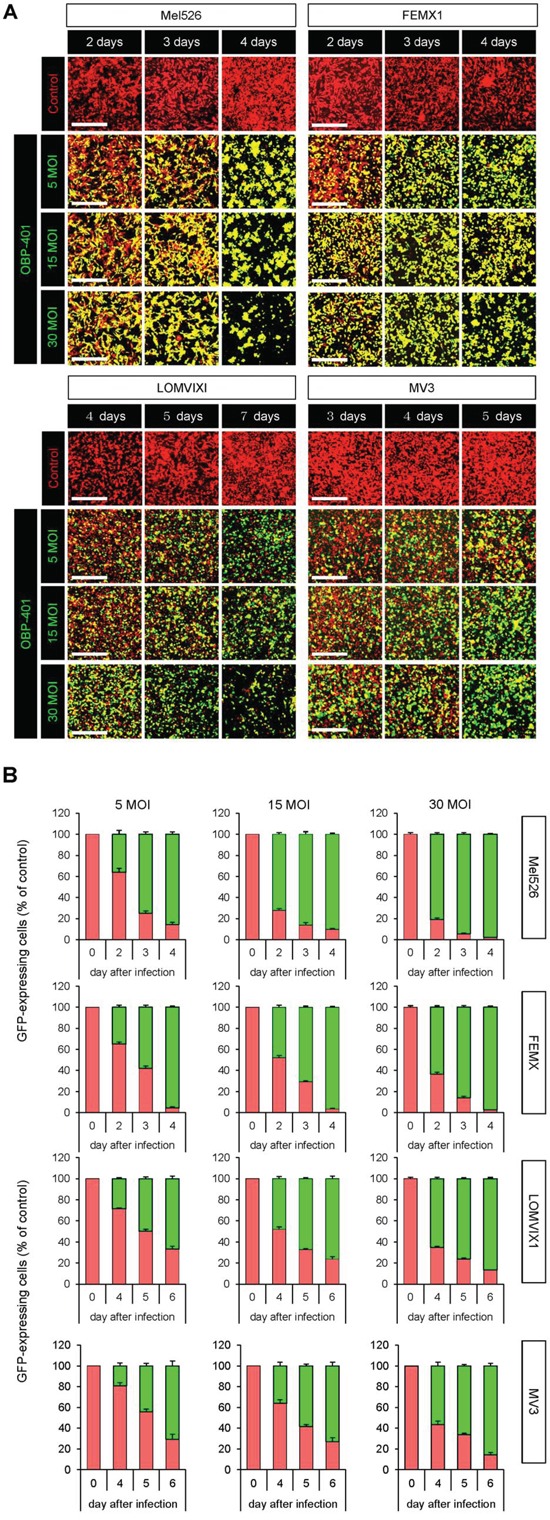
OBP-401 targets human malignant melanoma cell lines with GFP *in vitro* RFP-expressing human malignant melanoma cell lines, Mel526, FEMX1, LOMVIXI, MV3 were seeded on 6 well plates (1×10^5^ cells well). OBP-401 was added at the indicated multiple of infection (MOI) 24 hours after cell seeding. Images were acquired with an FV1000 confocal laser scanning microscope (Olympus). **A.** Time-course images of Mel526, FEMX1, LOMVIXI, MV3 cells 2, 3, and 4 days after infection with OBP-401, at indicated MOIs. **B.** Histograms show the frequency of GFP-expressing Mel526, FEMX1, LOMVIXI, MV3 cells at indicated days after infection with OBP-401. Data are shown as average ± SD. N = 5. Scale bars = 500 μm.

Time-course imaging demonstrated that OBP-401 labeled RFP-expressing Mel526, FEMX1, LOMVIXI, MV3 malignant melanoma cells with GFP (Figure 1A). GFP fluorescence, after OBP-401 targeting of Mel526, FEMX1, LOMVIXI, MV3 cells, became stronger each day from day 2 to day 7 (Figure 1A, 1B).

### Orthotopic malignant melanoma model

An orthotopic malignant melanoma tumor model was established with FEMX1-RFP cells implanted in the femoral skin where FEMX1-RFP cells produced a nodular tumor (Supplementary Figure S1A, S1B). The orthotopically-growing malignant melanoma cells invaded the quadriceps femoris muscle and metastasized to lymph nodes similar to the clinical course of melanoma (Supplementary Figure S1C). Tumor growth was visualized by RFP fluorescence (Supplementary Figure S1C).

### Bright-light surgery results in residual melanoma cells in the orthotopic model

**Figure 2:**
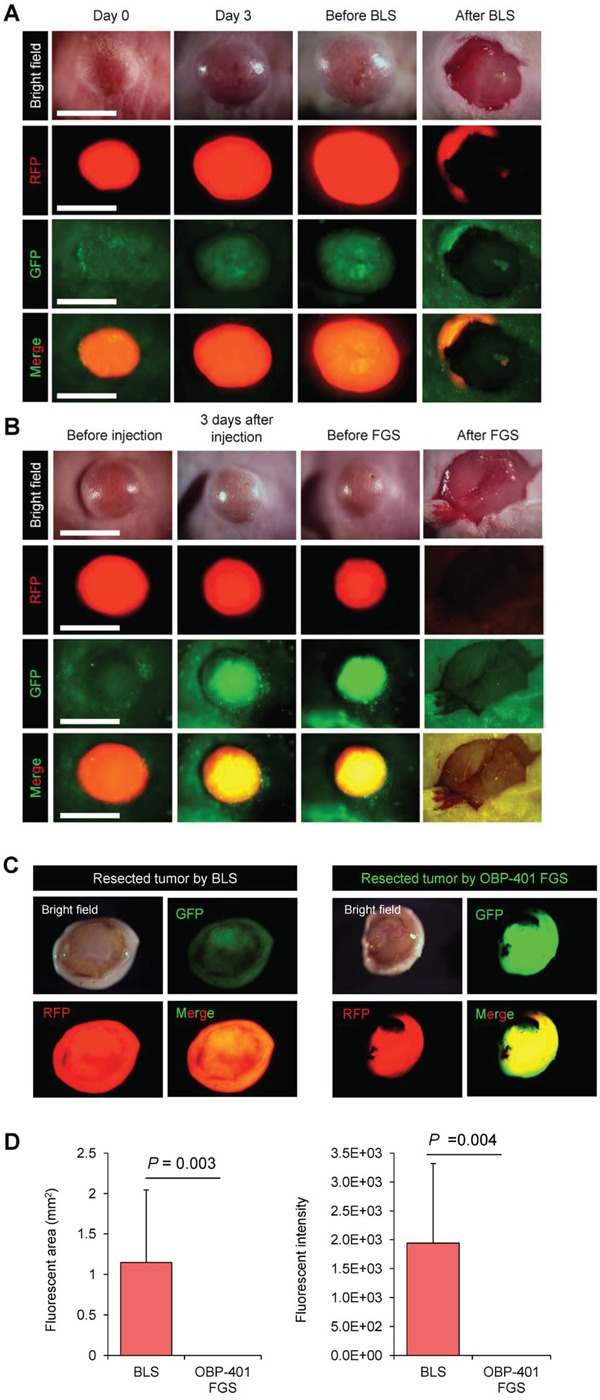
Comparison of OBP-401-targeted fluorescence-guided surgery with bright-light surgery for orthotopic malignant melanoma using a stationary imaging system RFP-expressing FEMX1 cells (5×10^6^) in 20 μl Matrigel (BD) were inoculated in the femoral skin of nude mice (5 weeks old). OBP-401 (1×10^8^ PFU) was injected intratumorally when tumors reached approximately 100 mm^3^ (6 mm diameter). **A.** Representative whole-tumor images of mock-infected REP-expressing orthotopic melanoma before and after bright-light surgery (BLS). **B.** Representative whole-tumor images of OBP-401-GFP labeled, RFP-expressing orthotopic malignant melanoma before and after OBP-401-based fluorescence-guided surgery (OBP-401 FGS). **C.** Representative whole-tumor images of a tumor resected by BLS and a tumor resected by OBP-401 FGS using the OV100 whole body imaging system. **D.** Bar graph shows the comparison of fluorescent area in the surgical bed after BLS or OBP-401 FGS (left). Bar graph shows the comparison of fluorescence intensity in the surgical bed after BLS or OBP-401 FGS (right). Fluorescent area and fluorescence intensity are calculated with ImageJ software. Data are shown as average ± SD. N = 12. Scale bars = 1 cm.

We performed bright-light surgery (BLS) on the orthotopic malignant melanoma model (Figure 2A, Supplementary Figure S2A). Due to inability to clearly visualize the tumor margins, extensive RFP-expressing malignant melanoma cells remained after BLS (Figure 2A, 2D).

### OBP-401-based fluorescence-guided surgery (OBP-401-FGS) of orthotopic melanoma using a stationary imaging system

The orthotopic tumor growing in the femoral skin (100 mm^3^, diameter; 6 mm) was resected 3 days after i.t. injection of OBP-401 (1×10^8^ PFU) (Figure 2B, Supplementary Figure S2B). OBP-401 conferred GFP fluorescence of the orthotopic melanoma which was sufficiently bright to perform complete resection using the OV100 whole body imaging system (Figure 2B). Tumor imaging showed that OBP-401 GFP labeling co-localized with tumor RFP fluorescence (Figure 2B, 2C). OBP-401-GFP-targeted FGS resulted in no detectable residual melanoma cells (Figure 2B, 2D).

### OBP-401-FGS of malignant melanoma using a hand-held portable fluorescence imaging system

**Figure 3:**
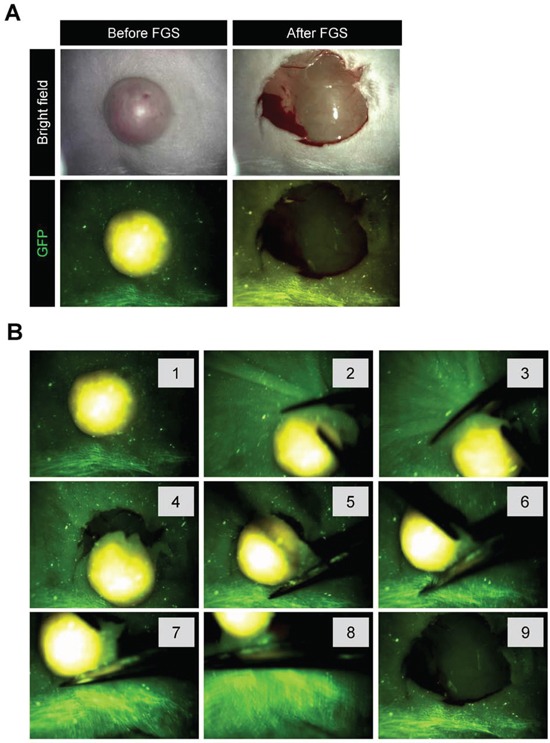
OBP-401 based FGS of melanoma using the Dino-Lite hand-held portable fluorescence scope **A.** Representative whole tumor images of OBP-401-GFP labeled, RFP-expressing orthotopic malignant melanoma before and after OBP-401-FGS using the Dino-Lite hand-held fluorescence scope. **B.** Step-by-step procedure for OBP-401 based FGS of malignant melanoma using the Dino-Lite hand-held-fluorescence scope.

**Figure 4:**
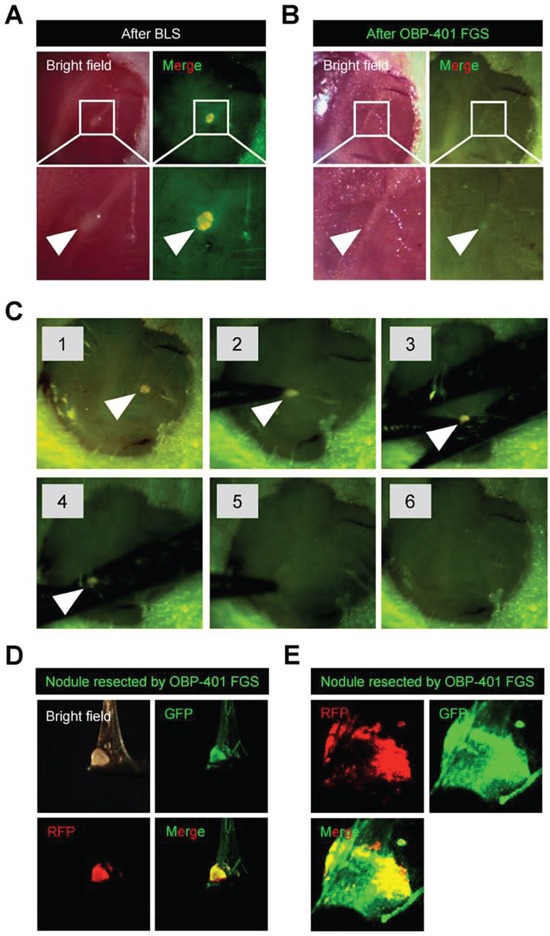
OBP-401 targeting visualizes residual malignant melanoma cells after BLS **A.** Representative high-magnification images of the surgical bed of orthotopic malignant melanoma after BLS. **B.** OBP-401 targeting enabled visualization of residual tumor after BLS and results in complete resection. Representative high-magnification images of surgical bed after FGS of malignant melanoma. **C.** Step-by-step procedure of OBP-401 based FGS of residual malignant melanoma after BLS using the Dino-Lite hand-held fluorescence scope. **D.** Representative images of resected malignant melanoma nodule by FGS. Images were acquired with the OV100. **E.** Representative single-cell level images of resected malignant melanoma nodule after FGS. Images were acquired with the FV1000 confocal microscope.

OBP-401-GFP targeting enabled the use of the Dino-Lite hand-held portable fluorescence imager [11] for FGS (Figure 3). OBP-401-GFP labeling for four days made the tumor margin much clearer than under bright-light (Figure 3A). Using the Dino-Lite, the tumor margin was clearly visualized enabling complete resection of the malignant melanoma (Figure 4B, Supplementary Movie S1). Fluorescence imaging showed that there were no residual cancer cells after OBP-401 FGS of malignant melanoma with the Dino-Lite (Figures 3, 4).

### OBP-401-FGS targets residual melanoma cells in the orthotopic model after BLS and enables complete resection

OBP-401 was intratumorally injected 3 days before BLS of FEMX1-RFP melanoma growing in the femoral skin (Supplementary Figure 3). OBP-401 enabled detection of the residual cancer cells at the single cell level in the surgical bed after BLS using a whole body imaging system (OV100) (Figure 4A). After OBP-401-FGS, there were no detectable residual cancer cells (Figure 4B, 4C). OBP-401 delineated the precise margins between cancer and normal tissue. OBP-401 enabled determination whether there were residual cancer cells at the single-cell level after FGS using confocal microscopy (FV1000) (Figure 4D, 4E).

### High-dose OBP-401 kills melanoma cells growing orthotopically *in vivo*, thereby enabling minimal surgery

**Figure 5:**
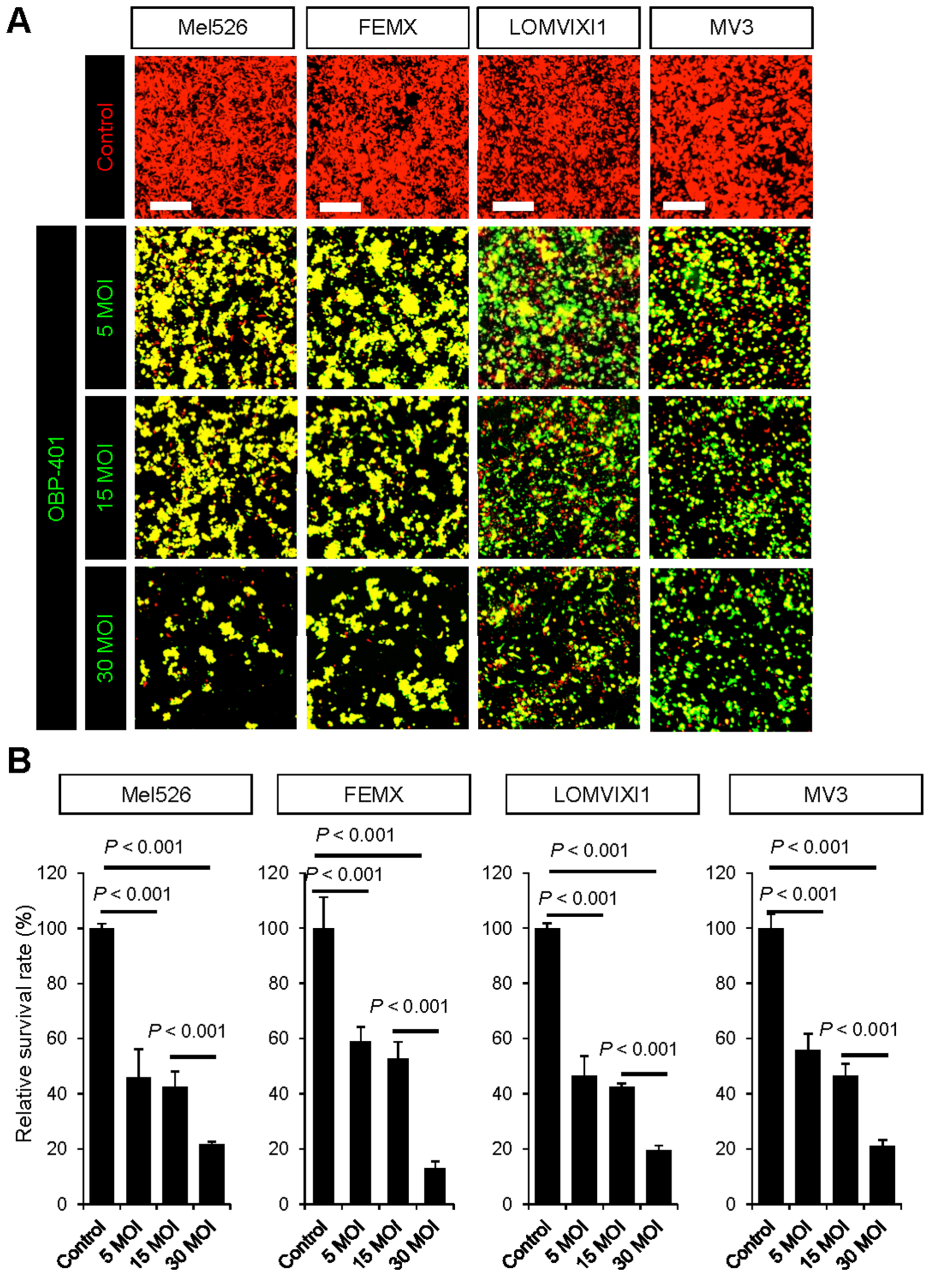
OBP-401 kills human malignant melanoma cell lines *in vitro* **A.** Representative images of RFP-expressing human malignant melanoma cell lines, Mel526-RFP, FEMX1-RFP, LOMVIXI-RFP, and MV3-RFP, 7 days after targeting with OBP-401 at a MOI of 5, 15, and 30. **B.** Bar graphs show the surviving fraction of Mel526-RFP, FEMX1-RFP, LOMVIXI-RFP, and MV3-RFP cells 7 days after targeting with OBP-401 *in vitro.* The number of live cells was counted. Data are shown as average ± SD. N = 5. Scale bars = 250 μm.

**Figure 6:**
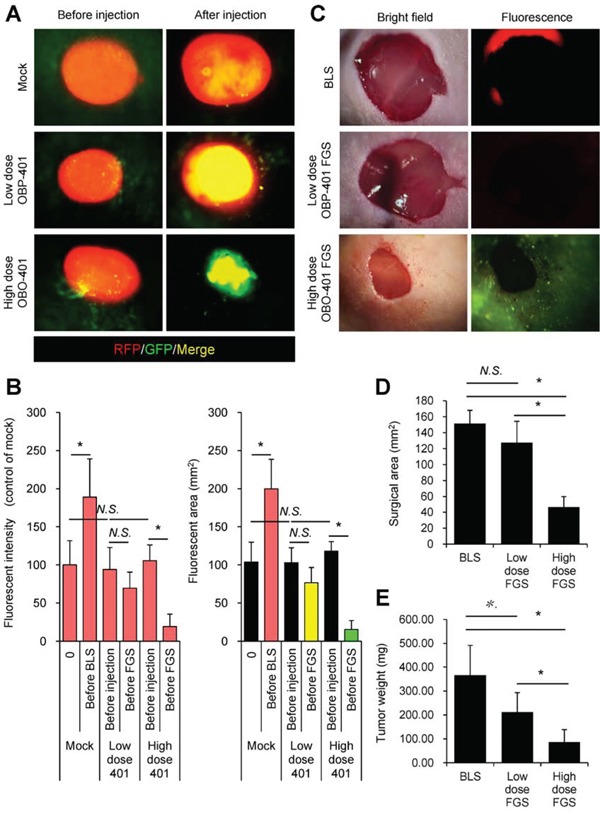
OBP-401 enables minimal precise surgery for orthotopic melanoma **A.** Representative whole-tumor images of orthotopic malignant melanoma before and after mock-infection or before and after i.t. administration of low-dose OBP-401 (1×10^8^ PFU) or high-dose OBP-401 (at 3×10^8^ PFU). **B.** Bar graphs show the fluorescence intensity of a control tumor and low-dose or high-dose OBP-401-targeted tumors (left). Fluorescent area is calculated with ImageJ software. Bar graphs show the fluorescence area of control tumor and low-dose or high-dose OBP-401-targeted tumors (right). Fluorescence intensity is calculated with ImageJ software. Data are shown as average ± SD. N = 10. **C.** Representative images of surgical area after BLS (upper) and low-dose (middle) or high-dose OBP-401-FGS (lower). Bright field images (left panels) and fluorescence images (right panels). **D.** Bar graphs show the comparison of surgical area after BLS, low-dose FGS, or high doe FGS. **E.** Bar graphs show the comparison of tumor weight after BLS, low-dose FGS, or high-dose FGS. Data are shown as average ± SD. N=10, *p<0.05.

High-dose OBP-401 targeted and killed Mel526-RFP, FEMX1-RFP, LOMVIXI-RFP, MV3-RFP cells in a dose-dependent manner *in vitro* (Figure 5). Therefore, we determined whether high-dose OBP-401 (2×10^8^ PFU)FGS enabled minimally-invasive surgery of melanoma compared with BLS or low-dose FGS. High-dose OBP-401 injected i.t. significantly reduced the size of tumors compared with untreated control tumors or low-dose OBP-401 (Figure 6A, 6B). The surgical area necessary for high-dose OBP-401 FGS was smaller than that for BLS or low-dose OBP-401 FGS (Figure 6C, 6D). The weight of tumor resected by high-dose OBP-401 FGS was less than that by BLS or low-dose OBP-401 FGS due to the cytotoxic effects of OBP-401 (Figure 6E).

### OBP-401 based FGS resulted in recurrence-free surgery

**Figure 7:**
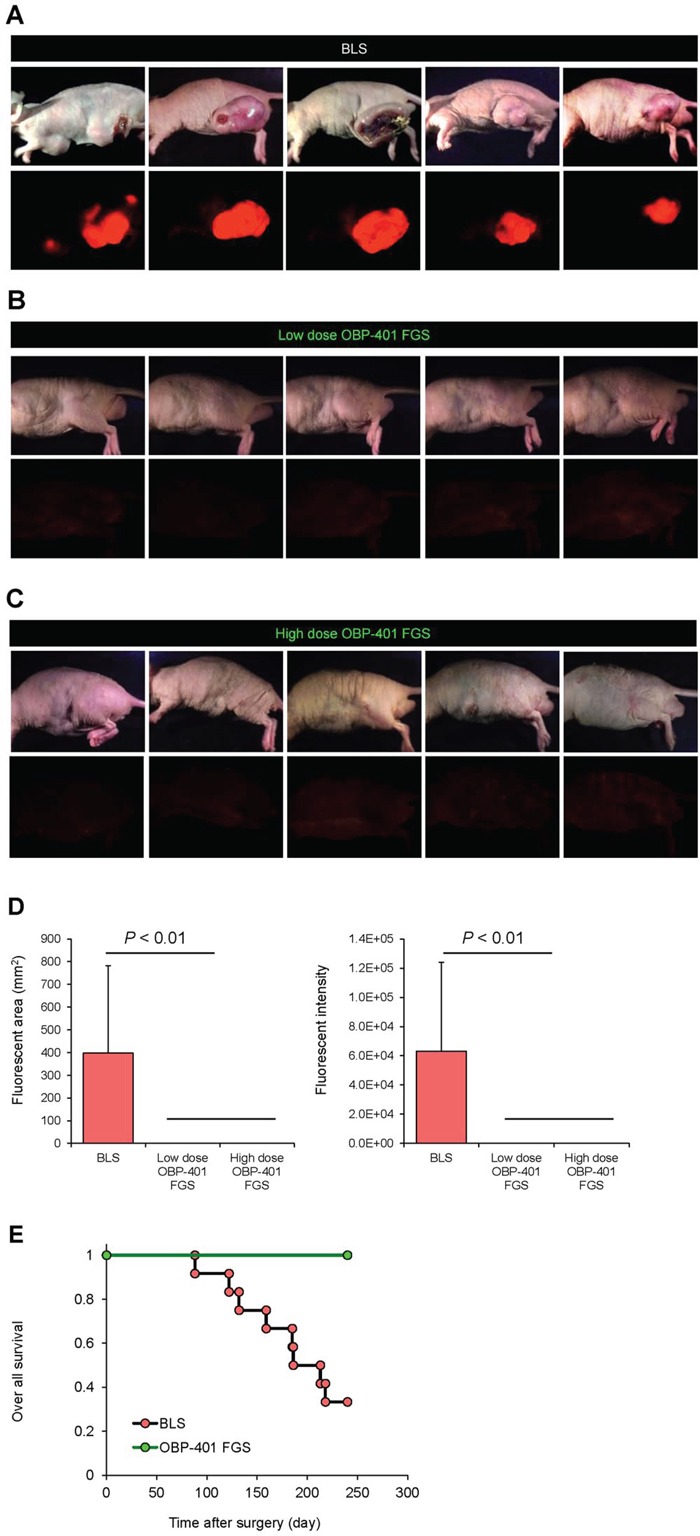
OBP-401-based FGS prevents local recurrence **A.** Representative whole-body images of orthotopic malignant melanoma 90 days after BLS. **B.** Representative whole body images of orthotopic malignant melanoma 90 days after low-dose OBP-401-FGS. **C.** Representative whole body images of orthotopic malignant melanoma 90 days after precise minimal FGS with high-dose administration of OBP-401. Upper panels in A, B, C: brightfield; lower panels: fluorescence. **D.** Comparison of fluorescent areas of recurrent tumors after BLS, low-dose OBP-401-FGS, or high-dose OBP-401-FGS (left panel). Fluorescent area is calculated with ImageJ software. Comparison of fluorescence intensity of recurrent tumors after BLS, low-dose OBP-401-FGS, or high-dose OBP-401-FGS (right panel). Fluorescence intensity is calculated with ImageJ software. Data are shown as average ± SD. N = 10. **E.** Kaplan-Meyer curves show the over-all survival after BLS or OBP-401-FGS.

Fluorescence imaging showed that eight of twelve mice that underwent BLS had RFP-expressing local recurrences (Figure 7A, 7D and 7E). In contrast, there was no local recurrence in twelve mice which received low-dose OBP-401-FGS (Figure 7B, 7D and 7E). Moreover, there was also no local recurrence in ten mice which received minimal surgery after high-dose administration of OBP-401 (Figure 7C, 7D and 7E) (Table 1).

**Table 1:** Tumor recurrence

Local recurrence	Positive	Negative
Conventional BLS	8	4
Low-dose OBP-401 FGS	0	12
High-dose OBP-401 FGS	0	10

*P = 0.001, **P = 0.003

Labeling tumors with OBP-401 for FGS has none of the weaknesses of non-genetic labeling, particularly loss of label over time and limited expression of the marker used for labeling. OBP-401 may be a general method for labeling tumors that express telomerase, which are the vast majority, that could have broad application for FGS.

A Phase I clinical trial of i.t. injection of OBP-301, the parent of OBP-401, in patients with advanced solid tumors was well tolerated [12]. Melanoma occurs intracutaneously, and therefore OBP-401 could be directly delivered and be tested in the clinic in the near future.

Concepts and strategies of highly-selective tumor targeting that were previously developed [13–17] can take advantage of adenoviral targeting of tumors, including tissue-selective therapy which focuses on unique properties of normal and tumor tissues [13, 15, 18]. OBP-401 can possibly overcome de-differentiation of a tumor leading to resistance to targeted chemotherapy, because the targeted protein or pathway may no longer be expressed [18], since OBP-401 does not depend on such targets [15], but depends only on telomerase expression which is expected to be stable in cancer cells. OBP-401 may also be effectively combined with teratogens which could selectively effect cancer cells that are dedifferentiated [14]. Since OBP-401 can decoy quiescent cancer cells to begin to cycle [19], OBP-401 could be effectively combined with agents which selectively target proliferating cancer cells [16], where normal cells are protected by agents which induce wild type p53 [17].

Other fluorescence methods of labeling tumors are also available. Fluorescence labeling at long wavelengths, such as near infrared (NIR), enables potential greater depth of visualization of the label, but such non-genetic methods will not label recurrent tumors [20].

## MATERIALS AND METHODS

### GFP-expressing telomerase-specific adenovirus

In OBP-401, the promoter element of the human telomerase reverse transcriptase (*hTERT*) gene drives the expression of E1A and E1B genes, linked to an internal ribosome entry site, for selective replication only in cancer cells. The *GFP* gene is driven by the CMV promoter inserted in OBP-401 [21].

### Cell line and cell culture

Red fluorescent protein-expressing human malignant melanoma cell lines Mel526-RFP, FEMX-RFP, LOMVIXI-RFP, and MV3-RFP (AntiCancer, Inc., San Diego, CA) were maintained and cultured in DMEM medium with 10% fetal bovine serum (FBS) and 5% penicillin/streptomycin.

### *In vitro* imaging

Time-course imaging of OBP-401-GFP labeling of melanoma cell lines was performed with an FV1000 confocal laser-scanning microscope (Olympus, Tokyo, Japan).

### Animal experiments

Athymic (*nu/nu*) nude mice (AntiCancer, Inc., San Diego, CA) were kept in a barrier facility under HEPA filtration. Mice were fed with autoclaved laboratory rodent diet (Tecklad LM-485, Western Research Products). All animal studies were conducted in accordance with the principles and procedures outlined in the National Institutes of Health Guide for the Care and Use of Laboratory Animals under Assurance Number A3873– 01.

### Orthotopic melanoma model

RFP-expressing FEMX1 cells (5 × 10^6^) suspended in Matrigel (20 uL)were inoculated into the left femoral skin of athymic nude mice (5-weeks old). Tumor progression was monitored by noninvasive fluorescence imaging (OV100) [22].

### OBP-401 based fluorescence-guided surgery (OBP-401-FGS)

All animal procedures were performed under anesthesia using s.c. administration of a ketamine mixture [10 μl ketamine HCl, 7.6 μl xylazine, 2.4 μl acepromazine maleate, and 10 μl PBS]. Orthotopic melanoma labeled with GFP by OBP-401 was observed with noninvasive fluorescence imaging using the OV100 Small Animal Imaging System (Olympus) [22]. OBP-401 FGS was performed using either the OV100 or a Dino-Lite hand-held fluorescence scope (Dino-Lite digital camera, AM4113T-GFBW Dino-Lite Premier; AnMo Electronics Corp., Hsinchu, Taiwan) [11]. After surgery, the presence of cancer cells was observed with the OV100 and Dino-Lite. If there were residual cancer cells, an additional resection was performed [7].

### Statistical analysis

Data are shown as means ± SD. For comparison between two groups, significant differences were determined using the Student's *t*-test. For comparison of more than two groups, statistical significance was determined with a one-way analysis of variance (ANOVA) followed by a Bonferroni multiple group comparison test. Pearson chi-square analysis was used to compare the rate of local recurrence between BLS and OBP-401-FGS. Statistical analysis for disease-free survival and over-all survival was performed using the Kaplan-Meier test along with log-rank test. Pearson chi-square analysis was used to evaluate the rate of local recurrence and lung metastasis between BLS and OBP-401-FGS. *P* values of < 0.05 were considered significant.

## SUPPLEMENTARY FIGURES



## Abbreviations

GFP: green fluorescent protein
RFP: red fluorescent protein
FGS: fluorescence-guided surgery
BLS: bright-light surgery
