# Supplementary material for: Adenoviral targeting of malignant melanoma for fluorescence-guided surgery prevents recurrence in orthotopic nude-mouse models
Source: Oncotarget. 2015 Dec 18;7(14):18558–72. doi: 10.18632/oncotarget.6670 (PMC4951309; doi:10.18632/oncotarget.6670)
Supplement: Supplementary file 1 [file oncotarget-07-18558-s001.pdf]

SUPPLEMENTARY FIGURES

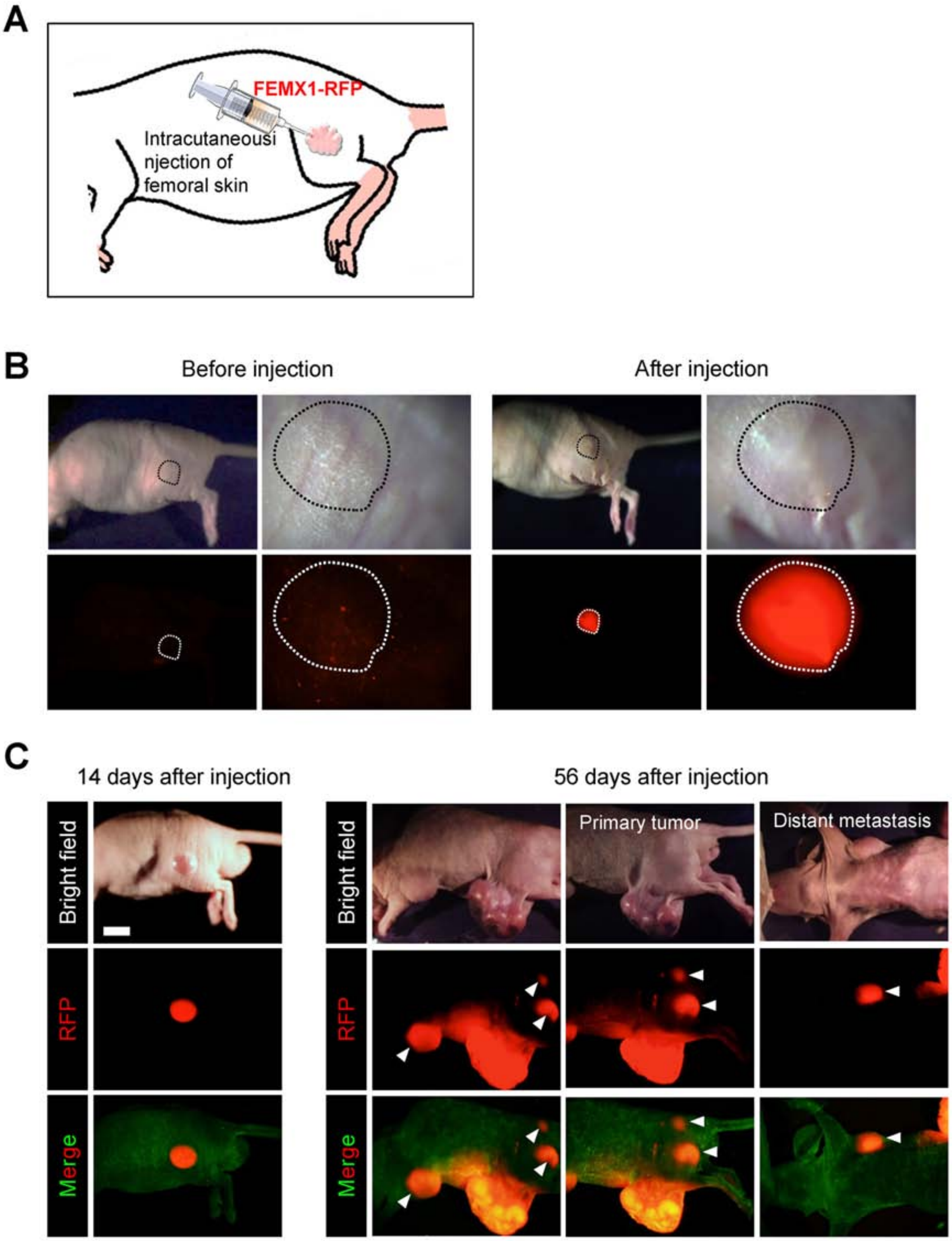

Supplementary Figure S1: Orthotopic malignant melanoma model. A. Scheme of orthotopic malignant-melanoma mouse model. **B.** RFP-expressing FEMX1 MM cells (5×10^6), suspended in Matrigel, were inoculated into the left femoral skin of athymic nude mice (5-weeks old). **C.** Representative images of orthotopic malignant melanoma 14 days cell after inoculation (left panel). Representative images of orthotopic malignant melanoma 56 days after inoculation (right panel). The RFP signal of the implanted tumors was visible outside the skin using a noninvasive whole-body fluorescence imaging system (OV100).

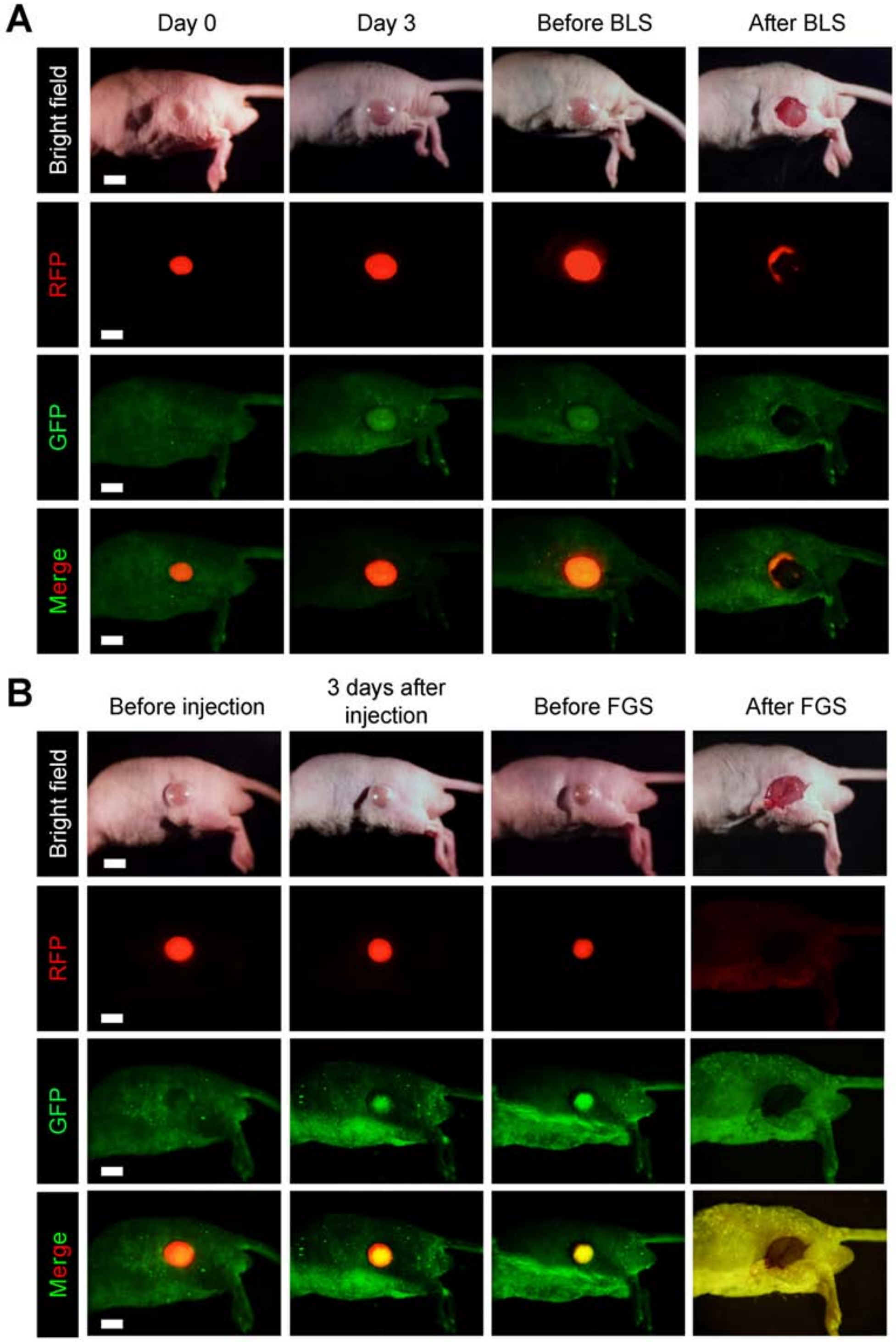

Supplementary Figure S2: Whole body imaging of bright light surgery and OBP-401 fluorescence-guided surgery of orthotopic malignant melanoma tumor. For fluorescence-guided surgery, OBP-401 was injected intratumorally at 1×10^8 PFU when tumors reached approximately 100 mm^3 (diameter; 6 mm). **A.** Representative whole body images of mock-infected orthotopic malignant melanoma before and after bright light surgery (BLS). **B.** Representative whole body images of orthotopic malignant melanoma before injection of OBP-401 and before and after OBP-401-based fluorescence-guided surgery (OBP-401 FGS). Images were acquired using a non-invasive whole-body fluorescence imaging system (OV100).

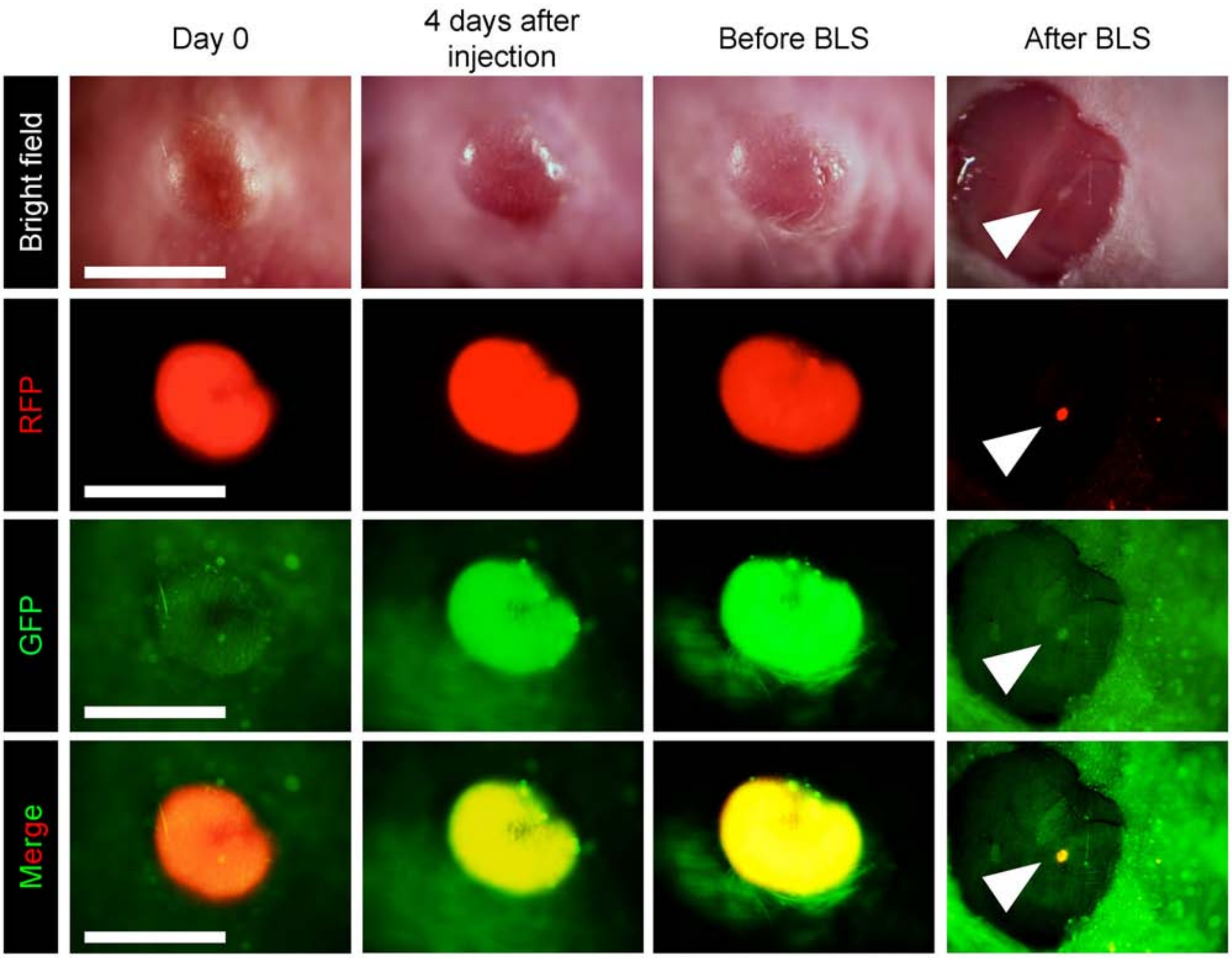

Supplementary Figure S3: BLS results in residual tumor in an orthotopic malignant melanoma model. To demonstrate OBP-401 visualization of residual malignant melanoma tissues after BLS in the orthotopic malignant melanoma model, FEMX1-RFP cells (5×10^6) in Matrigel were inoculated in the femoral skin of nude mice (5 weeks). OBP-401 was injected intaratumorally at 1×10^8 PFU when tumors reached approximately 100 mm^3 (6 mm diameter). The OBP-401-labeled orthotopic melanoma was resected under bright light, and then residual tumor was resected under fluorescence. Representative low-magnification images of orthotopic OBP-401 labeled malignant melanoma before and after BLS.

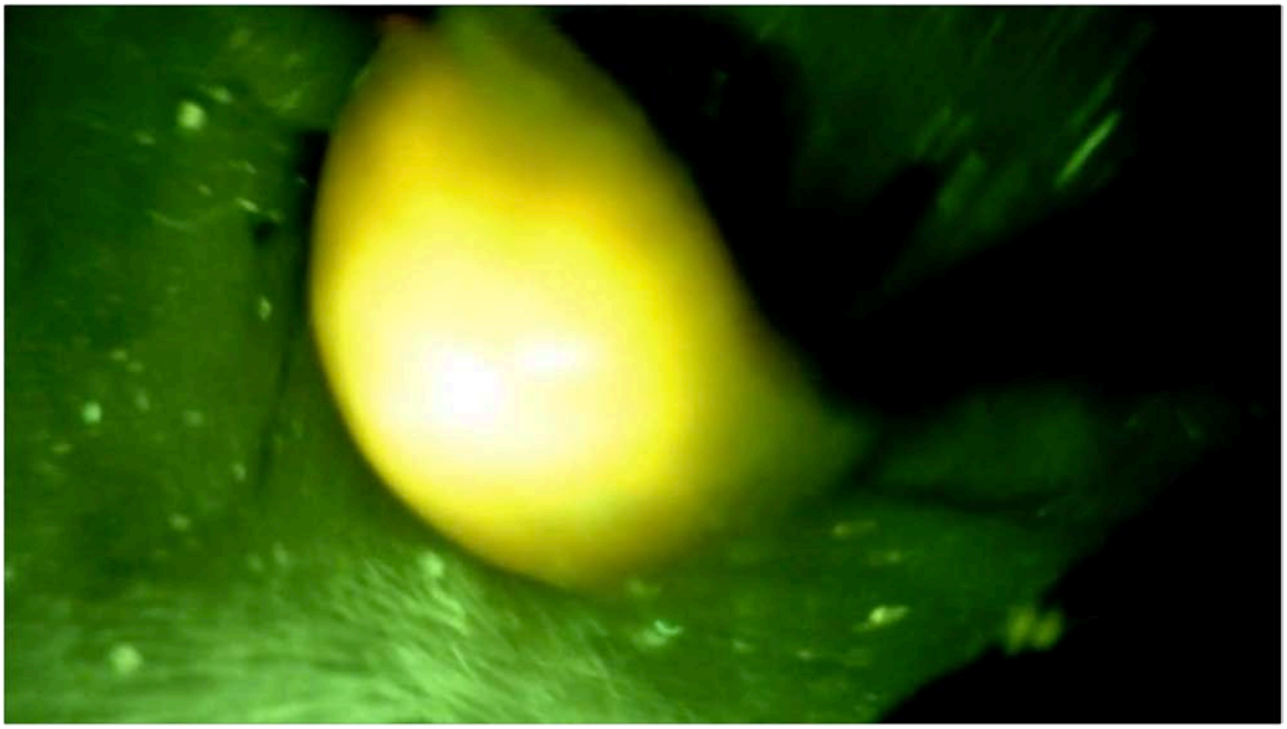

Supplementary Figure S4: Procedure for OBP-401-based fluorescence-guided surgery (FGS) of malignant melanoma. FGS was performed with the Dino-Lite hand-held portable fluorescence scope.
